# Physical explanations in evolutionary biology

**DOI:** 10.1007/s40656-025-00704-3

**Published:** 2025-11-28

**Authors:** Margarida Hermida, James Ladyman

**Affiliations:** 1 Philosophy Department GW, University of Salzburg, Franziskanergasse 1, 5020 Salzburg, Austria; 2 Department of Philosophy, University of Bristol, Cotham House, Bristol, BS6 6JL UK

**Keywords:** Autonomy, Biology, Evolution, Explanation, Physics, Reduction

## Abstract

The consensus in philosophy of biology seems to be that although nothing in biological systems is strictly incompatible with physical laws, biology is to a very great extent autonomous from physics. The main thesis of this paper is that, although biology is autonomous from the physical sciences in several ways, it is not explanatorily independent from physics. Physical explanations are pervasive and important in biology, including in evolutionary biology. The paper presents three case studies of physical explanations in evolutionary biology: a case of adaptation to pressure in deep-sea invertebrates, which illustrates how a physical parameter imposes selective pressures that organisms adapt to by changing their own physical properties; the case of water viscosity and the evolution of multicellularity, which shows how a global change in a physical parameter is thought to have triggered a macroevolutionary event; and the case of quantum tunnelling in respiration, which shows how quantum physics is relevant to the evolution of sex in eukaryotes.

## Introduction

One of the main philosophical issues about the biological sciences is “the relation between biology and physics” (Carnap, 1934, p. 18). This issue lies behind disputes about the autonomy of biology, organicism, reductionism and emergence in living systems, and the nature of life. The consensus in philosophy of biology seems to be that although nothing in biological systems is strictly incompatible with physical laws, biology is to a very great extent autonomous from physics. The main thesis of this paper is that, although biology is autonomous from the physical sciences in several ways, it is not explanatorily independent from physics. Physical explanations are pervasive and important in biology, including in evolutionary biology.

The question of whether biology is a legitimate science was once debated (Nagel, 1951; Smart, 1963) but is now beyond dispute. It is also indisputable that biology is autonomous from the physical sciences in the sense that it is a science in its own right rather than a branch of some other science, like particle physics and astrophysics are branches of physics. However more interesting claims about autonomy have also been made. In some of his writings on the autonomy of biology, Ernst Mayr had primarily in mind the idea that biology is *distinct* from the physical sciences in certain ways, due to peculiar features of living systems. These features include, especially, the *uniqueness and variability* of organisms which, unlike the entities studied in the physical sciences, form populations of non-interchangeable individuals, with variation among them being an important component of the explanation of evolutionary processes; the *complexity* of biological systems and their hierarchical organization; and the fact that biological systems depend for their functioning on the presence of vast amounts of *historically acquired information* (Mayr, 1985, 1996) including, but not limited to, genetic information. These features of biological systems are taken to motivate distinct methodologies that are unique to biology.

Mayr is surely right that biology as a whole is a distinct science, but the study of complex physical systems has shown that some of the features listed above are found beyond biology. Galaxies and solar systems are complex systems that also exhibit a degree of hierarchical organisation. Astrophysics studies huge populations of unique and variable stars, and though they do not evolve by natural selection the nature of later generation stars does depend on the nature of the previous generations. Some of the methods used in biology are also used to study non-living systems and vice versa.

Mayr was partly concerned with the question of “whether biology, after the refutation of vitalism, can be completely reduced to the laws and theories of the physical sciences” (1985, p. 53). The autonomy of biology is often taken to amount to the *irreducibility* of biology to physics. This is a fraught topic on which there is much disagreement. The next section discusses various senses of reduction that are relevant for investigating the relation between biology and physics, and argues that physical reduction, in the sense of providing physical explanations of biological phenomena, is often justified. Section 3 discusses the role of physical explanations in biology in general, and Sect. 4 considers in more detail how physical explanations are important in evolutionary biology and illustrates this with three case studies. Section 5 is a brief conclusion.

## Autonomy and reduction

Taxonomies of reduction standardly distinguish between explanatory and ontological reduction. Ontological reduction means reducing the *entites* of one domain to those of another domain, in this case by identifying token biological entities with token physical entities; the idea being that, in some sense, the objects and processes of biology are *nothing over and above* physical objects and processes. It is not exactly clear what this means, but it is usually taken to entail that there is nothing ‘spooky’ going on in living things; no vitalist residue that contradicts known physics. In this sense, Pradeu argues there is “full consensus” regarding the thesis of ontological reductionism, understood as the claim that “all biological processes are nothing other than physicochemical” (2018, p. 451). This does not mean that biological entities can, or should, be eliminated. On the contrary, a scientific realist should agree that the entities of biology are as real and causally efficacious as those of the physical sciences.

Explanatory reduction, in contrast, is a kind of epistemic reduction in which some phenomenon is explained in terms of some other, more fundamental phenomenon.^1^ It is this kind of reduction that is most often at stake in philosophy of biology, and it is the focus of this paper. There are several kinds of explanatory reduction. One of the most commonly discussed is the Nagelian notion of theory reduction, where the idea is that some theory of a ‘higher-level’ science is fully reduced to a theory of another, ‘lower-level’ science, if the former can be fully explained by the concepts and laws of the latter, given some bridge laws that ‘translate’ between the two. It is controversial whether, for example, classical Mendelian genetics has been reduced in this way to molecular biology (see, e.g., Kitcher, 1984; Waters, 1990; Schaffner, 1993), but it is implausible that all biological theories could be reduced, in this sense, to physical theories. More broadly, Fodor (1974) argued that the special sciences are autonomous from the more fundamental sciences because there is no one-to-one correspondence between the kinds of the special sciences and more fundamental science kinds. Often, the kinds found in the special sciences are multiply realizable, in that they can subsume vastly many physical realizers. Type physicalism must therefore be rejected, although token physicalism may still be accepted, since every token instance of a biological kind must have some physical realizer(s).

Nevertheless, multiple realization does not mean that *just about any* physical substrate could serve as the realizer of any biological function – on the contrary. A key made of brass will open the door just as well as one made of steel, provided they have the same shape; but one made of cotton candy will not, because it lacks the required tensile strength and rigidity. Similarly, specific chemical elements, molecules, and physical arrangements of matter are used by living systems for distinct functions due to their physical properties. Perhaps cell membranes do not have to be made of phospholipids, but in an aqueous environment there aren’t many alternatives to lipids that could fulfil the same function; perhaps DNA with different bases could be equally functional; but it probably could not fail to be some kind of long polymer.

Franklin (2022) has recently argued that the autonomy of the special sciences (and even much of physics) from more fundamental physics should be understood in terms of the states of a system at a higher scale being invariant with respect to some perturbations in variables at lower, more fundamental levels, and suggests that this kind of autonomy is compatible with reductive explanation. This kind of invariance seems to underlie some claims about the autonomy of biology; for example, when Mayr states that “disturbances at the level of elementary particles are ordinarily of no effect whatsoever at the higher levels of biological integration” (1985, p. 45). However, as Franklin notes, autonomy in this sense is a matter of degree, and biological systems are not fully invariant with regards to their physical realizers. Biology may be autonomous, but it is not free-floating, completely unconstrained by physics. And in fact, in some cases, disturbances at quantum scales *can* have highly relevant effects at higher levels of biological integration, as discussed in Sect. 4.3.

A more specific kind of explanatory reduction often discussed in biology is genetic and/or molecular reduction. Genetic reductionism is the thesis that genes can explain all the phenotypic features of an organism, including its morphology, behaviour, and even psychological properties in the case of humans (Sarkar, 1998, p. 174). This kind of reductionism is almost universally rejected, and rightly so, since it is well known that environmental factors play important roles in organismal development and can even regulate, promote, and supress gene expression.

Molecular reductionism is broader, insofar as it includes other macromolecules such as proteins; however, it is still unjustified because, regardless of how important the behaviour of molecules may be for the functioning of biological systems, some biological explanations require appeal to supramolecular features, such as membranes (Mitchell, 1961), or to the quantum behaviour of electrons (see Sect. 4.3). Part-whole reduction means explaining the functioning of a whole in terms of the functioning of its parts and their interactions, and is a standard scientific methodology which is also useful in biology. The ‘parts’ in question may or may not be physical parts and, when they are, they are usually not microphysical or molecular, but can be at many scales, including macroscopic parts. When the parts are physical objects, this kind of part-whole reduction corresponds to what Sarkar (1998) terms ‘strong reduction’ or ‘physical reduction’.

The kind of explanatory reduction to physics defended in what follows involves providing a physical explanation of a biological phenomenon–i.e. a phenomenon that occurs in and is characteristic of some biological entity or system, such as an organism or cell (Weber 2005, p. 25)–by the application of physical laws, theories, or principles. Importantly, this explanation can be at any scale and from any domain of physics (for instance, Newtonian mechanics, thermodynamics, quantum physics, etc.). Physical reduction in this sense is not only defensible, but has been very successful in biology. As Sarkar suggests, much of the confusion surrounding reduction in biology stems from conflating genetic and physical reductionism (1998, p. 14). The next section addresses the role of physical explanations in biology.

## The place of physical explanations in biology

Physical and chemical properties of biological systems, such as their mass, shape, physical structure, density, chemical composition, etc., are clearly relevant for the explanation of some biological phenomena. For example, the mass, density, and volume of an aquatic animal determine its buoyancy in water, and its mass and surface area determine its drag coefficient, both of which are important physical parameters for explaining fish swimming (Videler, 1993). Physical explanations of biological phenomena are particularly prevalent in ‘experimental biology’, i.e., those areas of biology primarily concerned with proximate explanations (roughly, the part of biology formerly known as ‘physiology’), including genetics, biochemistry, molecular biology, microbiology, neurobiology, and developmental biology (Weber, 2005). For example, the mechanism of the action potential, lock-and-key molecular receptors, the operation of motor proteins, chemiosmosis, or the dynamics of chromatin architecture, all involve physical laws and principles.

When it comes to evolutionary biology, however, the general view is that physical explanations are not very relevant; rather, what is needed is a historical explanation of how organisms came to acquire particular traits. Mayr argued that although a “strictly physicochemical explanation [may be found] at the cellular-molecular level” (2004, p. 36), “the physicochemical approach”, which he characterizes as the reductionist method of analysing the functioning of a system by dissecting it into its components, “is totally sterile in evolutionary biology. The historical aspects of biological organization are entirely out of reach of physicochemical reductionism” (Mayr, 2004, p. 71). This supposed inapplicability of physics and chemistry to evolutionary biology is due to the requirement, in biological explanations, of appealing to two kinds of causation: proximate and ultimate or evolutionary causation (Mayr, 1961), the latter being unique to biology. The idea is that for evolved biological systems, such as organisms, “a full explanation of all [their] phenotypic attributes (…) depends on both functional and evolutionary causality” (Bock, 2017, p. 76), and physics has no purchase on the latter:

All biological phenomena and processes are controlled both by proximate and by evolutionary causations. The biology of proximate causations can indeed, to a large extent, be reduced to chemistry and physics. Yet nothing in the realms of physics and chemistry is equivalent to the evolutionary causations that are controlled by the genetic programs of all organisms. (Mayr, 1996)

Weber, who defends a reductionist account whereby explanations in experimental biology are really physicochemical explanations of biological phenomena that rely on the explanatory force of principles from physics and chemistry, explicitly states that the intended domain of application of his account does not include evolutionary biology, classical genetics, or population ecology, all of which “have self-contained theories, law-like generalizations, and explanatory force of their own” (Weber 2005, p. 28). We have no intention of denying that these areas of biology feature theories, law-like generalizations, and have explanatory force; nor do we claim that all evolutionary explanations, for instance, are really physical explanations; this is evidently not the case. We do not propose physical explanations as *alternatives* to evolutionary explanations of the origin of organismal traits (D’Arcy Thompson 1917; see Gould, 1971 for critical discussion).

Evolutionary explanations are not completely invariant with respect to physics, because if physics had been different, evolutionary history would have been different too. However, we argue that physical explanations are directly relevant for evolutionary explanations. This does not mean that evolutionary biology can be fully reduced to physics, nor does it contradict Mayr’s claim that “no principle of historical evolutionary theory can ever be reduced to the laws of physics or chemistry” (2004, p. 79). But it does mean that *local* reductive explanations of evolutionary outcomes in terms of physical laws or principles are possible; that physical explanations are present throughout the biological sciences, including in evolutionary biology, rather than being restricted to ‘proximate’ causes and the functioning of biological mechanisms; and it means that physical explanations are essential in biology whereas the reverse is not the case.

The next section considers physical explanation in evolutionary biology in more detail focusing on three case studies.

## Physical explanations in evolutionary biology

There are at least five ways in which physical explanations are important in evolutionary biology: (1) physics *constrains* the space of biological possibilities, and thus set limits to what is evolutionarily possible; (2) physical laws and principles also *enable* the functioning of living organisms, and organisms *face selection* via their physical properties that confer them their functionality; (3) physical parameters of the environment impose selective pressures on organisms and lineages, leading to adaptation through cumulative directional selection; (4) physical perturbations involving global or very widespread changes in physical parameters have shaped macroevolution in various ways; and (5) physical laws governing the functioning of organisms can have important evolutionary consequences.

It is widely acknowledged that physics constrains what is biologically possible. Physical laws provide the background ‘rules of the game’ where ecology and evolution take place.

Every aspect of biology, from the energy budget of a hummingbird to the transpiration rate of a giant sequoia, is constrained by the physical basis of the natural world. (Taylor & Thomas, 2014, p. 31)

The laws of physics and the physical properties of materials out of which organisms are built, as well as their arrangement and complexity, set constraints on the functioning, development, and evolution of biological systems. For example, constraints imposed by the physical and chemical properties of CO_2_, as well as thermodynamic principles, explain limits to evolutionary optimisation of the enzyme RuBisCO, which catalyses the first step in the carbon dioxide fixation in plants (Tcherkez et al., 2006; Morange, 2011).

Biophysical constraints are most evident in biomechanics, where, for example, the movement of animals is largely governed by Newtonian mechanics, and aerodynamical principles are essential for explaining the shape of bird wings. Physical principles also set trade-offs that cannot be directly overcome–for example, a change in the shape of the skeletal component of a limb that increases the speed at which it can move always simultaneously reduces the force which it can exert (Maynard Smith et al., 1985); and biomechanical trade-offs related to feeding may explain some adaptive radiations, including the radiation of Darwin’s finches (Grant, 1986; Garland et al., 2022).

However, it is less often noted that physical laws and principles also enable the functioning of organisms, and thus play direct and indirect roles in ecology and evolution. In fact, organisms and their traits face selection via their physical properties which confer them their functionality. Consider any morphological trait which is under selection. An organism’s fitness is not a direct result of its genome, but neither is it a direct result of the phenotype. Rather, fitness depends on how the organism’s phenotypic features interact with the environment. In particular, *performance* on a particular task that is under selection (e.g. locomotion, feeding, etc.) mediates the relationship between structural variation and fitness (Arnold, 1983), and physical laws play a particularly important role in how morphology determines performance.

Thus, it is not only the case that the existence of physical explanations in biology is fully compatible with the fact that biologists also give functional explanations (Weber 2005, p. 41), the relation between the two kinds of explanation is a lot closer in two ways. First, the reason why some structure is able to fulfil a certain *causal role* function is due to its physical properties. Secondly, it is because of how its physical properties allow the structure to perform in a certain way on a task that is under selection that organisms with that structure undergo positive selection, and, therefore, given evolutionary time structure becomes an *aetiological* function, too.

Furthermore, physical parameters of the environment (and their variability) impose selective pressures on evolving populations and lineages. For example, temperature, pressure, pH, and other physical and chemical parameters limit the habitability of an environment and impose significant selective pressures on organisms to adapt to those conditions. The first case study below (Sect. 4.1) discusses how pressure has led to adaptations in the membrane phospholipids in deep-sea invertebrates. These adaptations are, in turn–unsurprisingly–also based on physical laws. Over time, the evolutionary response of lineages to environmental pressures results in the tweaking of physical features of organisms as they adapt to their environments.

Physical perturbations, involving global or widespread changes in physical parameters, have also shaped macroevolution (Jablonski, 2003). Most conspicuously, events such as asteroid strikes and extensive glaciations have often led to mass extinctions, but large-scale changes in physical parameters are also thought to have triggered important evolutionary events including, for example, a major evolutionary transition to multicellularity; this is discussed in the second case study below (Sect. 4.2). Different lineages would have responded differently to the change in the same physical parameter. Although it is recognised that intrinsic biotic factors may be involved in the way that different lineages respond to physical perturbation (Jablonski, 2003), in this case the different responses are hypothesized to be mainly due to physical factors which resulted in different groups of organisms experiencing different selective pressures when faced with a change in the same physical parameter.

Finally, physical laws governing the functioning of organisms can also have important macroevolutionary consequences. Although it is recognised that intrinsic physical factors, such as those involving biomechanical constraints on development and function, are important in evolution, it is primarily extrinsic ones that come to mind in the case of macroevolution. However, in the third case study (Sect. 4.3) we discuss how a physical phenomenon (quantum tunnelling) that is essential for respiration and photosynthesis may have played an important role in the evolution of eukaryotes, leading to extremely far-reaching consequences including the evolution of sex.

### Adaptation to pressure in deep-sea invertebrates

Temperature and pressure are physical parameters that can pose significant challenges to living organisms. High temperatures tend to destabilise biomolecules, and adaptation to very hot environments often involves structural changes to proteins that increase their stability at high temperatures (Berezovsky & Shakhnovich, 2005). Very low temperatures, on the other hand, cause lipid membranes to become less fluid and transition into a gel phase. The appropriate biological functioning of cell membranes depends on their physical properties, including their phase behaviour, degree of fluidity/viscosity, thickness, and rigidity, and these properties are determined by the molecular composition and curvature of the membrane (Ernst et al., 2016). Adaptation to cold temperatures thus tends to involve changes in lipid composition that increase membrane fluidity, a response known as homeoviscous adaptation.

High pressure also reduces the fluidity of lipid membranes, and can therefore drive similar adaptations as to cold temperature (Corkins et al., 2025). However, phospholipid compressibility is highly anisotropic, which means that pressure also has a strong effect on lipid shape (Winnikoff et al., 2024). For this reason, depth adaptation has also involved structural changes in lipid membranes that are distinct to those resulting from adaptation to the cold. In ctenophores–gelatinous marine invertebrates also known as comb jellies – adaptation to the deep-sea involved accumulation of the phospholipid plasmenyl phosphatidylethanolamine (PPE) in cell membranes, an adaptation that apparently evolved three times in separate ctenophore lineages (Winnikoff et al., 2024). These molecules have the most negative spontaneous baseline curvature of any phospholipid, which means that membranes with high levels of PPE are not viable at normal pressures. However, high pressure reduces this extreme curvature, such that the membranes are physiologically viable at depths to which these animals are adapted (Winnikoff et al., 2024).

This case demonstrates how a physical parameter imposes a selective pressure on organisms due to the changes it causes to the biophysical properties of an essential component of the organism, namely cell membranes. Since the functional properties of membranes depend on these biophysical properties, adaptation to the deep-sea environment has required structural changes in the lipid composition of membranes such that not only are membranes more fluid at high pressures and low temperatures, but they accumulate lipids with high spontaneous curvature that are able to maintain membrane shape at high pressure. Thus, organisms have adapted to a physical parameter by changing the physical properties of their membranes.

### Water viscosity as a trigger for the evolution of multicellularity

The macroevolutionary history of life on Earth is marked by crucial events known as major evolutionary transitions (Maynard Smith & Szathmary, 1997). The most important of these involved the evolution of novel hierarchical levels of organisation which correlate with sharp increases in organismal size. This was the case for the evolution of the eukaryotic cell and multicellularity. Although simple multicellularity is also found in bacteria, complex multicellularity is unique to eukaryotes, and evolved multiple times in several eukaryotic lineages during the Neoproterozoic era (1000 −541 Ma). Recent works suggest that very extensive glaciations, known as ‘Snowball Earth’ events, may have triggered the evolution of complex multicellularity by imposing a selective regime characterised by high water viscosity due to the low temperatures (Simpson, 2021).

The extent of the ‘Snowball Earth’ glaciations is disputed, with the hard Snowball scenario suggesting that there would have been 1 km of sea ice at the equator, whereas under soft Snowball or ‘slush-ball’ scenarios areas of open ocean would have remained near the equator. Even in the best case scenario, however, sea water temperature would have dropped to between − 4 and 4 °C across all latitudes and depths, resulting in a complete loss of warm habitats for most of 80 million years (Simpson, 2021). Temperature has a dramatic effect on the viscosity of water. The low temperatures and high salinities present in the Cryogenian era, regardless of the extent of glaciation, would have led to a doubling of water viscosity.

This is highly relevant because unicellular organisms are small (between 0.5 μm and 1 mm in length), which means that viscous forces dominate their environment. Although eukaryotes are much larger than bacteria, their Reynolds numbers are still well below 1, so a doubling of viscosity would have resulted in a significant impairment of their motility and feeding capability. The capacity of these small organisms to acquire nutrients depends on water diffusivity, density, and viscosity, which are environmental factors that organisms have no control over, but it also depends on the size or the organism and its velocity in the water. This suggests two possible evolutionary responses: evolving larger size and/or the capability to move faster.^2^ But it is hard for a tiny cell to move faster. Due to biophysical constraints, adding cilia or flagella does not increase motility, and increasing cell size also does not significantly increase velocity. It does increase encounter rate; however, the encounter rate scales with surface area whereas the organism’s metabolic rate scales with its volume; therefore, a nutrient deficit ensues (Simpson, 2021).

On the other hand, multicellularity allows organisms to increase in size faster than their metabolic demands increase. This is especially the case for hollow multicellular organisms morphologically similar to contemporary *Volvox*, but all forms of multicellularity are associated with sublinear scaling of metabolic rates with size (Crockett et al., 2024). Multicellular organisms are also able to attain higher speeds, thus increasing encounter rates and therefore their feeding capabilities. Furthermore, at Reynolds numbers between 0.1 and 50, encounter rates are higher than expected, due to compression of streamlines around the moving organism (Simpson, 2021). The size increase made possible by multicellularity would have enabled animals to escape the viscous environment of low Reynolds numbers in favour of a flow environment that was previously unexplored. Increasing cell numbers would then quickly have led to spatial structure, with internal cells being free from motility requirements, which enabled the evolution of division of labour, cell differentiation, and ultimately complex multicellularity.

This biophysical constraint hypothesis for the evolution of complex multicellularity is corroborated by modelling (Crockett et al., 2024), as well as by the dating of the evolution of multicellularity in multiple lineages to the Cryogenian (e.g. Bowles et al., 2024). The multiple contemporaneous transitions to multicellularity that occurred around this time strongly point to an environmental driver, but one that did not affect all lineages equally (Crockett et al., 2024). Biotic factors are known to condition the differential response of taxa to the same environmental perturbation. Among intrinsic biotic factors, Jablonski (2003) cites “body size, mobility, metabolic rate, physiological tolerance limits”. It is clear that these factors, however, are either physical in nature (e.g. body size), or strongly dependent on biophysical constraints (e.g. mobility; metabolic rate). Size, in particular, result in different groups of organisms experiencing different selective pressures in the face of a change in the same physical parameter–increased water viscosity. Some, such as bacteria, were confined to a diffusive ecology, and could only adapt by decreasing their size so as to reduce their metabolic needs in the face of lower diffusivity; whereas the comparatively much larger eukaryotes, at intermediate Reynolds numbers, had a novel evolutionary pathway open to them, via a modular organization that allowed them to reach larger sizes and speeds, and explore physical scales dominated by flow.

### From quantum tunnelling to sex

All complex life on Earth is eukaryotic. Eukaryotes originated from an endosymbiotic partnership between an ancient archaeon and a bacterium, with the latter evolving into the mitochondria that power our cells. The evolution of many complex eukaryotic traits seems to have been kickstarted by this endosymbiosis (Lane & Martin, 2010), and electron tunnelling in respiratory chains is thought to be crucial to the explanation of these important evolutionary outcomes.^3^

Organisms on Earth obtain energy via the process of chemiosmosis (Mitchell, 1961), a mechanism whereby electron transfer is coupled with the establishment of a proton gradient across a membrane. In both respiration and photosynthesis, electron transfer between redox centres provides the energy to pump protons across a membrane that is impermeable to them, generating a strong electrochemical potential. In much the same way that the water in a hydroelectric dam is only allowed to pass through the turbines, which allows the system to turn its potential energy into kinetic energy (and ultimately electricity), in mitochondrial membranes the protons are only allowed back through the ATP synthase, which uses the energy from this disequilibrium to physically rotate and produce ATP from ADP and inorganic phosphate, to supply the cell’s energy needs.

The process of electron transfer relies on quantum tunnelling. Electrons are quantum particles, understood as both material particles and waves, and can be described by a wave function, which is governed by the Schrödinger equation. When there is a potential barrier, the wave function is *dampened*, but if the barrier’s width is small enough, there is still a small amplitude of the wave function behind the barrier. This means that, for small enough potential barriers, there is a nonzero probability that the electron will ‘tunnel’ through the barrier even if there is no sufficient energy to overcome the barrier classically (Trixler, 2013).

It is through this process that, in respiration, electrons ‘jump’ from one redox centre to the next adjacent centre that is not already occupied. The distance between redox centres is therefore crucial, because quantum tunnelling only occurs consistently over distances of less than 14 Å. An increase of 1 Å in distance decreases the rate of electron transfer 10-fold (Lane, 2015, p. 241). Therefore, the respiratory complexes must be shaped just right for respiration to carry on at an appropriate rate. Indeed there is evidence that:

Natural selection at the molecular level acts on electron transfer protein design principally through the selection of distances of 14 Å or less between redox centres to assure adequately fast tunnelling down of the electron transfer chains, and even smaller distances, typically 7 Å or less, to assure adequately fast electron transfer with multi-electron catalytic clusters, where there are barriers to single-electron oxidation and reduction. (Moser et al., 2006)

However, in eukaryotes, an important complication arises. Although mitochondria are no longer separate organisms, but bioenergetic organelles, they still have their own DNA.^4^ This is surprising because there are strong evolutionary pressures for mitochondrial genes to be transferred to the nucleus, and indeed most have been–it is more efficient, allows centralised control to the nucleus, and protects DNA against mutation caused by free radicals of oxygen. Yet all eukaryotes with oxygen respiration have mitochondrial DNA. This is due to the need for certain genes to be physically located in the mitochondria to control respiration locally and allow the respiratory machinery to quickly respond to changes in electron flux, oxygen availability, ATP/ADP ratio, etc. (Lane, 2015). The co-location of crucial genes for respiration in the same compartment as their gene products facilitates direct regulatory coupling (Allen, 2002).

The result of this is that, in eukaryotes, some protein subunits of the respiratory complexes are coded by nuclear genes, and others by genes in the mitochondria. A fine balance between nuclear and mitochondrial genomes must therefore be maintained for respiration to proceed smoothly. Since an increase of only 1 Å in the gap between adjacent redox centres will slow down electron tunnelling by an order of magnitude, any slight mismatch between mitochondrial and nuclear genomes is likely to lead to a slowing of electron transfer and a rise in free-radical leak, which has indeed been observed (Lane, 2011).

Since eukaryotic cells rely on mitochondria for their bioenergetic needs, and due to the need for the continued existence of mitochondrial genomes, a tight co-adaptation of mitochondrial and nuclear genes is essential for eukaryotic life. This co-evolution of the two genomes is thought to drive many unique aspects of eukaryotic evolution, including sex with recombination and the existence of two sexes with uniparental mitochondrial inheritance (Hill, 2019).

Although bacteria can exchange genes through conjugation, sexual reproduction is unique to eukaryotes. It involves reciprocal gene exchange, chromosome recombination, and meiosis followed by fusion of haploid gametes to produce a diploid individual. Recombination is the crucial aspect of sex–it separates linked genes, which allows them to face natural selection individually, rather than as an entire chromosome, which makes it easier for favourable genes to be positively selected, and detrimental ones eliminated. This is important because mitochondrial DNA (mtDNA) mutates faster than nuclear DNA (nDNA) and has no recombination, so mutations constantly accumulate; enhanced selection at gene level reduces the negative effects of this mutational erosion, through compensatory co-evolution of nuclear genes. In other words, sex allows the nuclear genome to keep up with mtDNA mutational erosion.

A separate aspect of sexual reproduction is anisogamy, i.e. the existence of two sexes or mating types with distinct gametes. This evolutionary strategy does not seem prima facie advantageous, seeing as it immediately excludes half the population as potential mates, thus increasing the cost of mate search–a particularly significant problem for unicellular marine organisms, where anisogamy first evolved. However, only a mating system with two sexes enables uniparental inheritance of mitochondria. The transmission of mitochondria via one parent only prevents heteroplasmy, which would give rise to natural selection within the individual–otherwise, any mutation that promoted mitochondrial replication would be favoured by selection, even at a cost to the organism. This is the best combination of traits for dealing with mtDNA mutational erosion:

The optimal situation for efficient purging of deleterious mitochondrial alleles is to have little variation in the mt genotypes within an individual and substantial variation between individuals. (Hill, 2019)

It is generally thought that quantum processes are not directly relevant for biological explanations–only indirectly, insofar as quantum physics is responsible for the stability of matter, chemical reactions, etc. Indeed,

many scientists (…) accept that quantum mechanics must, at a deeper level, be involved in biology; but they insist that this role is trivial. (…) [S]ince the rules of quantum mechanics govern the behaviour of atoms, and biology ultimately involves the interaction of atoms, then the rules of the quantum world must also operate at the tiniest scales within biology–but *only* at those scales, with the result that they will have little or no effect on the scaled-up processes important to life. (Al-Khalili & McFadden, 2014, p. 37)

It is true that most biological processes take place at scales far above the quantum scale and, therefore, physical laws prevalent at those scales (e.g. gravity, fluid mechanics, etc.) are likely to be more relevant to the explanation of many biological phenomena. However, the phenomenon of electron tunnelling in respiration is not only essential to the explanation of a nanoscale mechanism; if the above hypothesis is true, this quantum effect also has far reaching consequences for the evolution of all complex life on Earth, as the need for co-adaptation between nuclear and mitochondrial genomes, which has driven the evolution of sex, can be traced back to the requirement for adequate positioning of protein subunits in respiratory complexes in order to maintain distances between redox centres small enough for electron tunnelling to take place.

## Conclusion

Although biology is an autonomous science in important ways, it is not explanatorily independent from physics. We have characterised the physical explanation of a biological phenomenon as a kind of reductive explanation that is distinct from genetic, molecular, and microphysical reduction, and consists in the application of laws, theories, and principles from any part of physics. It is uncontroversial that there are such physical explanations in biology; however, they are mainly thought to be restricted to proximate explanations of how living things work, and not very relevant for evolutionary biology. We have argued that physical explanations are in fact pervasive across all areas of biology, and are particularly important for explanations of evolutionary outcomes. We presented three case studies of physical explanations in evolutionary biology to support this claim. The case of adaptation to pressure in deep-sea invertebrates demonstrates how a physical parameter (pressure) imposed a selective pressure that organisms adapted to by changing the physical properties of their membranes; the case of the evolution of multicellularity in the Cryogenian exemplifies how a global change in a physical parameter triggered a macroevolutionary event; and, the case of quantum tunnelling in respiration shows how quantum physics is relevant to the explanation of the evolution of sex and other unique features of eukaryotes.

## Data Availability

Not applicable.

## Footnotes

1 For Moritz Schlick, “[s]cientific knowledge consists in the explanation of one set of phenomena in terms of another” (Schlick 1925/1953, p. 523).

2 A third evolutionary response is to become smaller, so as to minimise metabolic needs (Simpson, 2021). This is a viable option that has been followed by some eukaryotic lineages, although it implies encroaching on a niche already occupied by bacteria, which have several advantages over eukaryotes when it comes to miniaturisation.

3 Eukaryogenesis is widely considered to be a major evolutionary transition that occurred only once in the evolutionary history of life on Earth. For a dissenting gradualist view, see e.g. Booth and Doolittle (2015).

4 The same is true of chloroplasts in plants.
